# The influence of climate change on mental health in populations of the western Pacific region: An umbrella scoping review

**DOI:** 10.1016/j.heliyon.2023.e21457

**Published:** 2023-11-08

**Authors:** Aikaterini Vafeiadou, Michael J. Banissy, Jasmine F.M. Banissy, Julian P.T. Higgins, Guy Howard

**Affiliations:** a Department of Psychology, Goldsmiths, University of London, London, UK; b School of Psychological Science, University of Bristol, Bristol, UK; c Department of Population Health Sciences, University of Bristol, Bristol, UK; d Cabot Institute, University of Bristol, Bristol, UK

**Keywords:** Climate change, Mental health, Well-being, Western Pacific region

## Abstract

The Western Pacific Region (WPR) is on the front line of climate change challenges. Understanding how these challenges affect the WPR populations' mental health is essential to design effective prevention and care policies. Thus, the present study conducted an umbrella scoping review that examined the influence of climate change on mental health in the WPR, using review articles as a source of information. Ten review articles were selected according to eligibility criteria, and the findings were synthesized according to the socio-economic status of the countries identified: Australia, the Republic of Korea, the Philippines, Vietnam, the Pacific Islands (broadly), and China. The findings revealed that each country and sub-region has its own unique profile of climate change-related challenges and vulnerable populations, highlighting the need for specific approaches to mental health care. Specifically, the influence of climate-related challenges differed according to populations' region (e.g., rural populations), demographic characteristics (e.g., age and gender), culture (e.g., traditional tights to land), and employment (e.g., farmers and fishers). The most frequently reported mental health outcomes in response to climate change-related challenges such as droughts, floods, storms, tornadoes, typhoons, and climate-related migration were the decline in mental well-being and the increase in post-traumatic stress disorder symptoms. In addition, using the GRADE framework for assessing the certainty of the findings, we identified that the number of articles discussing associations between a given climate change challenge and a mental health outcome was overall limited. Based on our findings and findings on a global scale, we identified several key research gaps in WPR and provided recommendations for future research and policy strategies.

## 1 Introduction

### 1.1 How can climate change influence mental health?

Climate change has been referred to as "the biggest global health threat of the 21st century" [1]. Climate change health threats include the global rise in temperature, changes in precipitation patterns, and more frequent extreme weather events [2]. Moreover, emerging research suggests that the impact of climate change extends beyond physical health and has a devastating effect on mental health [[3], [4], [5], [6], [7], [8], [9], [10], [11], [12]].

The way that climate change exerts its impact on an individual's mental health is multifaceted [5,11]. First, an individual may experience a traumatic event directly via acute climatic exposure (e.g., intense precipitation, king tide) or chronic climatic exposure (e.g., rapid sea level rise, prolonged droughts). Climate change may also indirectly influence the individual's mental health via socio-political changes such as changing an individual's socio-economic status (e.g., decreased farming during droughts, migration due to sea-level rise) and via more general physical health threats (e.g., malnutrition). [5,11]

Social representations of climate change and personal ideology may also influence an individual's mental health by inducing "climate anxiety". This refers to the adverse emotional reactions stemming from an awareness of climate change [13]. New linguistic terms have been created in the past years to describe an individual's climate-related thoughts and feelings [12,14]. For instance, "eco-anxiety" is commonly understood as the emotional response individuals have, encompassing feelings of distress, apprehension, and unease when faced with the pressing challenges of global climate change and accompanying environmental decline [14]. Another term "solastalgia" describes the psychological distress induced due to change or loss of homeland environment [12]. The development of our climate-related vocabulary reflects the increasing concerns about individuals' mental health.

### 1.2 What is mental health?

Defining mental health is essential for understanding the relationship between climate change and mental health. Mental health relates to how we feel, think, and act in everyday life and can be further categorized into a) non-clinical conditions and b) clinical conditions [[15], [16], [17]]. Table 1 summarizes the non-clinical and clinical mental health conditions of interest in this paper.

**Table 1:** Non-clinical and clinical mental health outcomes that are relevant to the current study are presented in this table. The type of measurement, its purpose, and its level (individual or community) are identified for each mental health outcome.

	Mental Health	
	Non-clinical conditions	Clinical Conditions
**Measurement**	Subjective qualitative or quantitative methods. Quantitative tools are usually used for research purposes using published scales in the literature according to researchers' choice.	Subjective qualitative and quantitative methods. Subjective reports are assessed with a clinical interview and an established clinical quantitative measure widely accepted for clinical use.
**Purpose**	Research purpose	Clinical diagnosis by medical professionals. Indication of clinical diagnosis for research purpose.
**Individual level**	Economic well-being Mental well-being: - Evaluative well-being - Emotional well-being Social well-being	Post-Traumatic Stress Disorder (PTSD) symptoms Depression symptoms Anxiety symptoms Acute Stress Disorder symptoms
**Community level**	Community well-being	_

Non-clinical conditions are commonly grouped under the umbrella term "well-being." Although there is not a unanimous definition of well-being, there is a broad consensus that it encompasses subjective reports about an individual's life satisfaction, feelings or mood, cognitive abilities, economic productivity, and the degree to which someone feels a purpose and meaning in life [[18], [19], [20], [21], [22]]. Well-being definitions that consider an individual's ability to engage meaningfully in society emphasize personal freedoms, self-efficacy, human agency, and dignity [21,23]. It is also vital to consider cultural nuances; for instance, indigenous populations often intertwine well-being with their connection to nature and cultural identity [21,24]. In the context of climate change policy-making, recognizing these intricate facets of well-being is characterized as crucial to guide the formation of strategies that strive to be both economically efficient and equitable [21].

We report below the key well-being categories relevant to the present study and define them according to prior literature. These categories refer to well-being from an individual or community point of view.

1. ***Economic well-being:*** refers to an individual's capacity to sustain income and cover basic survival needs [25]. In the present study, we describe a decline in economic well-being when individuals report increased financial concerns, financial stress, decreased income or economic instabilities, increased depts, job insecurity, loss of property or housing, and reports of unemployment as consequences of climate change-related events.
2. ***Mental well-being:*** includes evaluative and emotional well-being [18,19]. In the present study, we describe a decline in mental well-being when at least one of these well-being measures is influenced by climate change-related events.
  a. Evaluative well-being: refers to life satisfaction. In the present study, we consider there to be a decline in evaluative well-being when individuals report a decrease in life satisfaction due to climate change-related events.
  b. Emotional well-being: it refers to affect, positive and negative emotions. In the present study, we consider a decline in emotional well-being when there are reports of individuals feeling helplessness, sadness, anxiety, fear, aggression, stress, or low mood due to climate change-related events. We also consider climate-related sleep disturbances as a mediating factor that could lead to a decline in emotional well-being, as suggested by the literature [26].
3. ***Social well-being:*** refers to the sense of belonging and contributing to a community and relates to feelings of loneliness [27,28]. In the present study, we consider a decline in social well-being when individuals report decreased feelings of connection to traditional values or cultural identity, or feelings of loneliness and isolation in response to decreased frequency of social gatherings and climate change-related events.
4. ***Community well-being***: While social well-being focuses on individual experiences within a community, community well-being emphasizes the health and vitality of the community as a collective unit. Communities are formed by people with shared values and intentions. Community well-being thus refers to the mechanisms that allow community members to improve/maintain aspects of their individual well-being, and it is tightly linked to members' social well-being. These mechanisms include a range of opportunities for support and social interactions, such as economic, social, environmental, cultural, and political initiatives. [29] Communities' well-being also relates to the notions of "social connectedness" or "social cohesion" to indicate how tight the bonds between its members [30]. In the present study, we consider a decline in community well-being to be when there is a reported decrease in community-wide social events or gatherings, or individuals express dissatisfaction with the local support systems, services, or community-driven initiatives, or when there is a perceived weakening of bonds or cohesion among community members. Thus, community wellbeing emphasizes how individuals evaluate their communities rather their own feelings and emotions [29].

The persistently low levels of one or more well-being categories may place individuals at high risk of developing a clinical mental health condition [[31], [32], [33], [34]]. Clinical mental health conditions, in contrast to subjective well-being reports, are identified by medical professionals using clinical interviews in combination with well-established quantitative measures such as the Diagnostic and Statistical Manual (DSM; commonly used in the USA) or the International Classification of Diseases (ICD; commonly used in the U.K. and by the World Health Organization). Mental health researchers may also use those measures to report an indication of clinical mental health symptoms in the population. In both cases (medical or research purpose), the number and intensity of symptoms that negatively impact an individual's life (e.g., persistent sadness, stress) are judged to identify if the individual suffers from a mental health condition. Those symptoms can influence personal, educational, occupational, and social aspects of an individual's life. The current study focuses on the symptoms of post-traumatic stress disorder, depression, anxiety, panic disorder, and acute stress disorder among the different mental health conditions. According to the ICD-11th version [35], these conditions are described as.

1 ***Post-Traumatic Stress Disorder (PTSD):*** exposure to a traumatic (horrific or life-threatening) event or a series of traumatic events may induce PTSD symptoms that persist for several weeks. The symptoms include 1) re-experiencing the event(s) even after its cessation via vivid flashbacks, memories, or intrusive thoughts 2) avoidance of situations and people that may place the individual in a high possibility of re-experiencing the event(s) via the mechanisms mentioned in (1) 3) perception of heightened threats such as hypersensitivity and adverse reactions to unexpected noise.
2. ***Depressive Disorder (DD) or Depression:*** it is one of the mood disorders. It can take many forms depending on the number of depressive episodes and the severity of depressive episode's symptoms that may persist over two weeks. The depressive episode symptoms include persistent depressed mood and loss of interest in previously enjoyable activities. Other symptoms that might also appear persistent include guilt, hopelessness and worthlessness, sleep and appetite disturbances, a decline in energy levels and fatigue, difficulty in concentration, psychomotor retardation or agitation, and suicide ideation.
3. ***Generalized Anxiety Disorder (GAD) or Anxiety:*** it is characterized by persistent anxiety for several months that could be described as more general apprehension or excessive worry over everyday life concerns such as family life, occupational, household, and financial concerns. Other symptoms include difficulty concentrating, motor restlessness, muscular tension, irritability, and sleep disturbance.
4. ***Panic Disorder (PD):*** it is distinguished by sudden occurrences of panic attacks (episodes marked by strong feelings of fear or anxiety and physical symptoms like elevated heart rate) and ongoing worries about experiencing them again.
5. ***Acute Stress Disorder (ASD):*** it is characterized by parodic cognitive, emotional, somatic, and behavioural symptoms in response to a life-threatening or horrific event that decays after a few days. Symptoms may include anxiety, negative emotions, confusion, and social isolation.

The current study reports clinical mental health outcomes more commonly for research than clinical purposes. In addition, the literature reports concerns about the cross-cultural validity of the clinical diagnosis tools developed in western countries, as they may not be relevant for clinical diagnosis in non-western countries [36]. Thus, to account for the above observations, we will refer to clinical mental health outcomes as clinical symptoms rather than clinical conditions (e.g., depressive symptoms and not depression) to avoid incorrect judgments of a clinical diagnosis.

Additionally, there is evidence linking sleep with emotional well-being and specific clinical symptoms [26,35,37,38]. Growing evidence also points to the increasing occurrence of sleep disturbances attributed to factors like rising temperatures and climate-change-related phenomena such as tornadoes and floods [39,40]. Given these factors, our study focuses on sleep as a pivotal physical health factor that can impact mental health. While a significant body of evidence supports the positive association between mental and physical health [41,42], an in-depth exploration of this relationship is beyond the scope of our current research.

### 1.3 Which populations are at risk of declining mental health due to climate change?

Climate change affects every country across the globe. However, to date, climate change's impact on mental health (or health more broadly) varies across different populations. Specific populations appear to be at higher risk of declined mental health than others, depending on their socio-economic status and their residence's geographical location.

Individuals with low socio-economic status who live in low and middle-income countries (LMICs) and areas that are more impacted by climate change seem to be at higher risk of declined mental health [7,36,43,44]. LMICs’ vulnerability to climate change relates to their limited capacity to offer economical and health support in response to climate change threats. LMICs have limited resources, weak infrastructure, and health services to cope with climate-related disasters and increased damage to the local and national economies when affected by climate change-related events. Consequently, populations in LMICs are more likely to face climate-related poverty and limited mental health support and are less likely to be covered by insurance. [36,44,45] In addition, individuals with low socio-economic status, irrespective of the country of residence, rely more on agriculture and natural resources for food and employment. This reliance can place them at a greater risk for malnutrition, unemployment, and home displacement when landscape changes result from climate change [45]. The above observations collectively indicate risk factors for declined economic, mental, social, and community well-being in LMICs and populations with low socio-economic status.

When assessing the impact of climate change on the different geographical regions, the Western Pacific Region appears on the frontline of climate change [46]. World Health Organization (WHO) identifies 37 WPR countries covering Oceania, the eastern half of the Eurasian landmass, and the eastern part of South-East Asia [46]. The WPR is experiencing increased intensity, frequency, and heterogeneity of climate change events. For instance, the sea level rise in WPR is recorded as higher than the global average [47].

However, the climate change events that each country in the WPR suffers more from seem region-specific. For instance, Australia suffers more from increased temperatures and prolonged droughts, positioning rural populations (e.g., farmers) at high risk of financial concerns and unemployment [[48], [49], [50], [51], [52]]. Pacific Islanders face the loss of coastal areas and changes in marine systems due to rapid sea level rise and ocean warming, forcing Islanders to face surviving challenges and forced migration [12,[53], [54], [55], [56], [57], [58]]. China – a country with rapid urbanization, seems to face diverse climate change events such as high temperatures, precipitation, floods, and sea level rise [59]. Similar diversity in climate change events appears in the Philippines and Vietnam, which suffer particularly from typhoons (also called cyclones), floods, and droughts, with sea level rise being a greater concern in Vietnam [46,60]. Finally, the Republic of Korea seems to suffer more from high temperatures and heatwaves [61].

### 1.4 The aim of the present study

The present study is an umbrella scoping review that aims to identify the influence climate change may exert on mental health in populations of the World Health Organization Western Pacific Region. Specifically, this study focuses on climatic events expected to increase in frequency and intensity due to rising global temperatures and climate change, such as floods and droughts. Non-climate-related natural disasters, such as earthquakes, are out of scope. In addition, the evidence of causality between the studied climatic events and mental health outcomes is evaluated. Finally, strategies for prevention and care and the research gaps are identified.

## 2 Methods

### 2.1 Search strategy

Database searches were conducted in six databases (PubMed, EMBASE, Web of Science, Scopus, PsycINFO, CINAHL) in October 2022. The search strings were constructed according to three themes; climate change keywords, mental health keywords, and geographical keywords. The keywords were created following an initial review of articles that discussed the global influences of climate change on mental health and through discussions with the research team. In addition, article type and language filters were used to identify review articles written only in English (see Supplemental Table 1 for a detailed description of the database search strings).

### 2.2 Eligibility criteria

Review articles that followed a screening selection process against eligibility criteria (systematic reviews, scoping reviews, meta-analysis) in English were included without restriction on publication date. In addition, articles were included if they discussed the direct or indirect influence of climate change on mental health in populations of at least one of the thirty-three countries in the WHO Western Pacific Region [62] and were grouped by socio-economic status. Table 2 presents a detailed description of inclusion and exclusion criteria.

**Table 2:** Scoping review exclusion and inclusion criteria.

	Inclusion Criteria	Exclusion Criteria
Article type	*Review type articles:* systematic reviews, scoping reviews, and meta-analyses published in journals.	Non-peer-reviewed articles, Grey literature, Commentaries and responses to reviewers/editors.
Regions	Articles that studied at least one of the following Western Pacific regions were included. *High-Income Countries (HICs):* Australia, Brunei Darussalam, Cook Islands, French Polynesia, Guam, Nauru, New Caledonia, New Zealand, N Mariana Islands, Japan, Palau, Pitcairn, Republic of Korea, Singapore. *Low to upper-middle-income countries of Pacific Island states (LICs to UMICs):* Fiji, Kiribati, Marshall Islands, Micronesia, Niue, Papua New Guinea, Solomon Islands, Samoa, Tokelau, Tuvalu, Vanuatu, Wallis & Futuna. *Low to mid-income countries of Continental Asia (LICs to MICs):* Cambodia, Lao PDR, Mongolia, Philippines, Viet Nam. *Upper middle-income countries of Continental Asia (UMICs):* China, Malaysia	Articles that refer to global or mixed population patterns without including studies of at least one of the inclusion criteria regions. Articles that refer to global patterns, including at least one country of WPR but without separating observations for that country.
Climatic exposures	*Climatic changes:* Temperature variations, heat, drought, sea level rise, rainfall, flood, coastal storms, glacial melting, decreased air quality, an increase of extreme weather events, and any natural disasters that are described as more intense due to climate change events.	Natural disasters that are not considered to be influenced majorly by climate change, such as earthquakes.
Mental Health Outcomes	*Mental health:* Post-traumatic stress disorder (PTSD), mood changes, depression, anxiety disorders, psychological distress, increased anxiety, increased worry, increased aggression, increased suicide rates, and the decline in any well-being category. *Hospital admission*s: increased admissions that evaluate mental health (time series analysis of clinical records). *Sleep*: worsened sleep quality that impacts mental health (intermediate outcome). *Community well-being:* decline in community well-being and social cohesion.	Hospital admissions that do not consider patients' mental health condition. Sleep outcomes that do not relate to mental health.
Relationship between Exposures and Mental Health Outcomes	*Direct/indirect influence* of climate change on mental health outcomes. *Interventions* evaluated for regulating the relationship between climate change and mental health outcomes.	Articles that discuss climate change interventions (policies and strategies of prevention and care) but do not directly evaluate mental health outcomes. For example, articles discussing how to improve health services to respond better to climate change-related issues but do not evaluate how this improvement may impact individuals' mental health outcomes.

### 2.3 Articles selection process

The articles’ characteristics (e.g., title, authors, DOI number) found from the database searches were compiled in an excel file and processed with a customized R script to identify duplicates based on the articles' DOI numbers. Duplicates were also evaluated manually. After removing the duplicates, two researchers screened articles independently (KV and JMFB) against the eligibility criteria (see Table 1). In case of disagreements, the issues were discussed until reaching a mutual agreement.

**Fig. 1:**
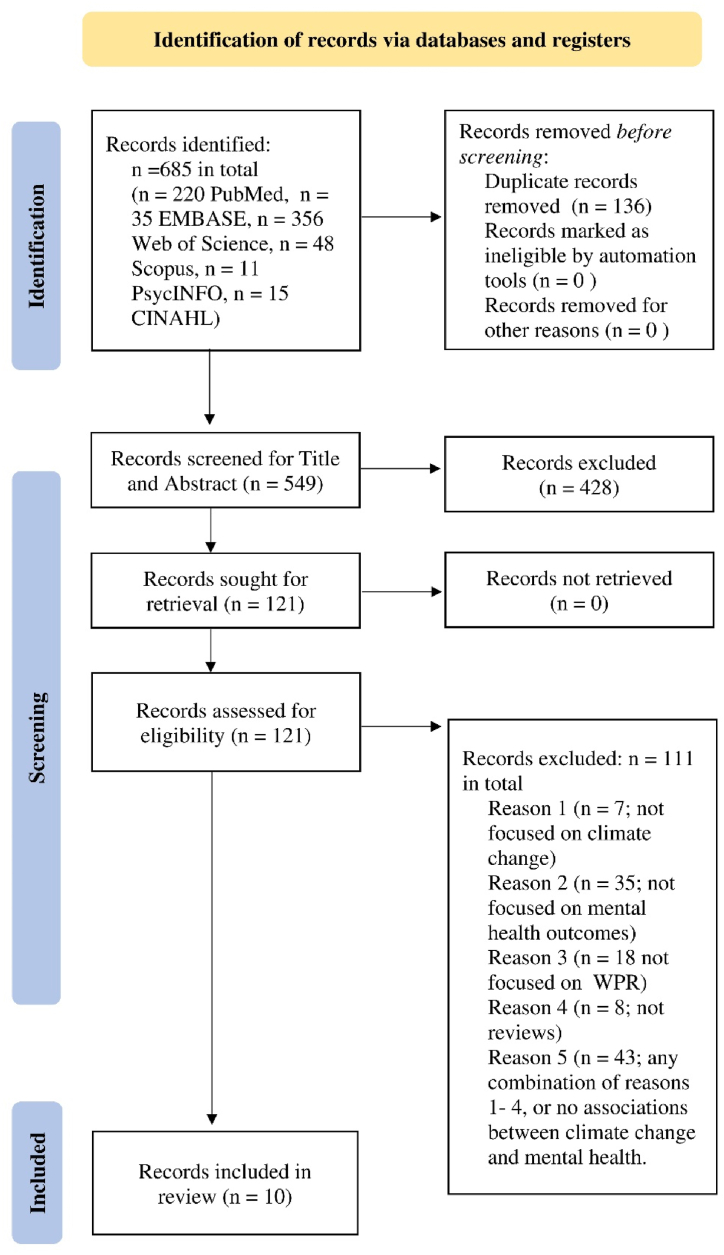
PRISMA flow diagram shows the number of identified records, the screening process, and the selected records (review articles).

The screening was conducted in two steps. First, titles and abstracts were examined by KV and JMFB. Review articles were retrieved if the title and abstract indicated that the article related to all the following themes: climate change or climate change-related events, any health outcomes that could be associated with mental health, and Western-Pacific or Global regions that could have studied WPR. The full-text records were then assessed by KV for their specificity to climate change, mental health, and WPR countries according to the eligibility criteria. Articles that discussed direct or indirect causality between climate change events and mental health in populations of at least one WPR country were included in the review. The findings of each screening step are presented in a Preferred Reporting Items for Systematic Reviews and Meta-Analysis flow diagram (PRISMA; Fig. 1).

### 2.4 Information synthesis, analysis & evaluation

The information from the selected articles was synthesized using a narrative approach with categorical descriptors [63] of the studied region(s), population type, climatic event(s), and mental health outcome(s). For this purpose, a Microsoft Excel form was developed to include descriptors for each study and aid visualizations of the articles’ characteristics.

## 3 Findings

### 3.1 Characteristics of the selected articles

**Fig. 2:**
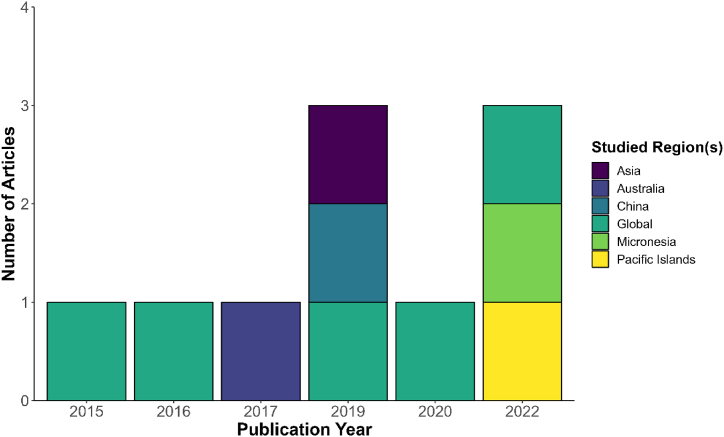
Bar plot showing the publication year of the selected articles and their studied region(s).

In our scoping review, n = 10 review articles were included. Most articles studied global regions (n = 5; these were included because they reported findings for at least one of the WPR regions of interest). Fig. 2 details the publication year and studied regions of the ten articles. See Supplemental Table 2 for a detailed description of the articles’ characteristics.

The abstracts of the ten selected articles were used to generate a Word-Cloud using the "wordcloud2" R package providing a visual illustration of the main themes explored within these articles. Before creating the final Word-Cloud the text underwent a text mining procedure using the "tm" R package. First, all words were transformed to lowercase and English punctuation and numbers were excluded. When the number of blank spaces was n > 1 the spaces were replaced by single blank spaces. In addition, meaningful words for the current review (e.g., "droughts") were inspected for derivatives and plurals. Singular and plural words were replaced by their singular version (e.g., "drought"). Derivatives (e.g., "flooding") were replaced by their noun (e.g., "flood"). Lastly, we inspected the words’ frequencies. Words that showed high frequency (f > 3) and were not meaningful in terms of their qualitative description of the relationship between climate change and mental health were excluded (e.g., verbs: will). The words "climate" (n = 26), "change" (n = 22), "mental" (n = 39), and "health" (n = 32) were excluded. This procedure resulted in n = 559 words in total. The Word-Cloud was generated using the 200 most frequent words.

Our analysis identified the following five words as the most frequently occurring, and these were interpreted within the context of the key articles’ findings:

Suicide (n = 13): This term appeared predominantly in discussions about the negative impacts of droughts on rural Australians. There was a particular emphasis on older rural males and farmers, pointing to a concerning trend within this group.

Australia (n = 12): A significant portion of the articles sourced their findings from this region, highlighting Australia's importance in the research scope.

Rural (n = 12): The research discussed the experiences and challenges of rural populations, mainly in Australia, with some references to Asia.

Farmer (n = 11): Articles prominently discussed the difficulties faced by farmers in Australia, especially during drought periods.

Communities (n = 10): In the selected articles, the term "community" referred to a broad range of groups studied. This predominantly included communities dependent on marine resources, such as fishers and others like Pacific Islanders, situated in coastal areas vulnerable to climate change impacts. Due to these climate-change challenges, these communities faced considerable challenges concerning food security, health, and the preservation of cultural practices. The experiences of Aboriginal communities in Australia were also given particular emphasis in response to climate-change phenomena. A common thread running through these articles highlighted communities' indispensable role in nurturing various aspects of well-being.

**Fig. 3:**
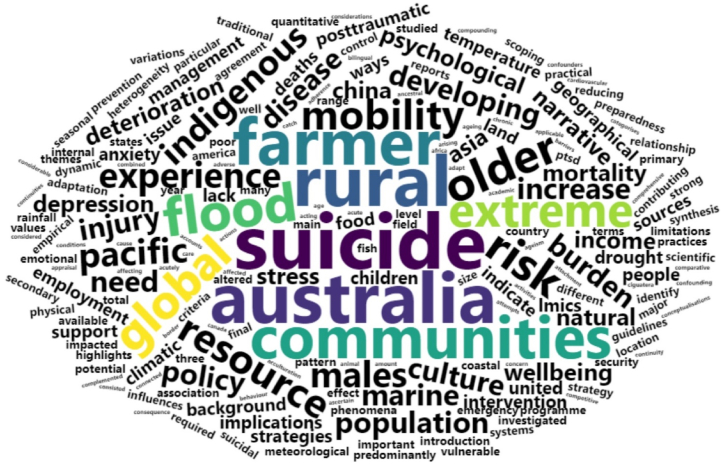
The World Cloud shows the 200 most frequent words from the abstracts of the selected articles. The more frequent the words, the bigger their font size.

For a detailed graphic representation of the top 200 most frequent words derived from the abstracts, refer to Fig. 3.

### 3.2 Certainty of findings

The certainty of the findings was evaluated using a modified version of GRADE [64]. Findings were assigned a Grade of 4 at the start and then downgraded considering five factors (see Table 3 for a detailed description of the quality assessment).

1*.Risk of bias:* Were there biases in the reviews when investigating the link between climatic events and mental health outcomes? We critically assessed potential measurement and methodological biases. Thus, we examined whether the review articles incorporated qualitative and quantitative research approaches. Additionally, we evaluated how thoroughly the reviews accounted for external factors, such as covariates, which could influence their findings. We also scrutinized the potential bias from omitted outcome data, notably in cases where referencing original studies was essential for a deeper understanding of the results.
2. *Imprecision:* How much evidence did the review(s) include? We examined the number of original articles reported in the review article(s) that investigated a given relationship between climate change and mental health.
3. *Inconsistency:* Is there evidence of variable effects? We examined if the findings show mixed or inconclusive findings when evaluating the relationship between climate change and mental health.
4. *Indirectness:* Do the review(s) and their included studies address our questions of interest? We examined the degree to which the review articles report direct effects between climate change and mental health.
5. *Publication bias:* Are we likely to be missing evidence? We did not examine this aspect as all review articles followed a selection process of their included studies considering eligibility criteria.

**Table 3:** Quality evaluation of the impact of climate change-related events on mental health outcomes per population type and studied region.

Climatic Event	Mental health Outcome	Studies	Quality of Evidence (GRADE)	Population type	Region
**Drought**	Decline in economic well-being	[52,65]	Low^a^ ^●●○○^	Farmers	Australia
**Drought**	Decline in mental well-being	[52,65]	Low^b^ ^●●○○^	Farmers	Australia
**Drought**	Decline in economic, social, mental, and community well-being	[66]	Very Low^c^ ^●○○○^	Aboriginals	Australia
**Flood**	Increased substances use: tobacco, alcohol, medication	[67]	Very Low^d^ ^●○○○^	No sample selection criteria	Australia
**Typhoon**	Post-Traumatic Stress Disorder (PTSD) symptoms	[68]	Very Low^e^ ^●○○○^	Elementary School students	Republic of Korea
**Typhoon**	Post-Traumatic Stress Disorder (PTSD) symptoms, Acute stress, Depression symptoms	[44,68]	Very Low^f^ ^●○○○^	Children and adolescents	Philippines
**Typhoon**	Post-Traumatic Stress Disorder (PTSD) symptoms, Major Depressive Disorder symptoms, Panic Disorder symptoms, General Anxiety Disorder symptoms	[36]	Very Low^g^ ^●○○○^	No sample selection criteria	Vietnam
**Pacific climate-related population mobility**	Decline in economic, social, mental, and community well-being	[53,58]	Moderate^h^ ^●●●○^	Dislocated Pacific Islanders (internal or cross-border mobility) & Pacific Islanders' subsistence fishing communities	Pacific Islands (origin of the population)
**Flood**	Post-Traumatic Stress Disorder (PTSD) symptoms	[44,59] [36,67,68]	High ^●●●●^	No sample selection criteria or adolescents or children	China
**Flood**	Anxiety symptoms	[59]	Very Low^i^ ^●○○○^	No sample selection criteria	China
**Typhoon**	Post-Traumatic Stress Disorder (PTSD)	[59]	Very Low^j^ ^●○○○^	Adolescents and patients with a clinical diagnosis of PTSD	China
**Tornado**	Post-Traumatic Stress Disorder (PTSD)	[44]	Moderate^k^ ^●●●○^	Adolescents	China
**Tornado**	Depression symptoms	[44]	Very Low^l^ ^●○○○^	Adolescents	China
**Storm**	Post-Traumatic Stress Disorder (PTSD)	[44]	Very Low^m^ ^●○○○^	High school students	China

a Risk of Bias: There were reported only qualitative data. Original articles were extracted. Indirectness: Review articles focus on general mental health risk factors in farmers and not specifically during drought.

b Risk of Bias: There was reported no statistical analysis on suicide rates. Original articles were extracted. Indirectness: Review articles focus on general mental health risk factors in farmers and not specifically during drought.

c Risk of Bias: There were reported only qualitative data. Inconsistency: There were mixed patterns about age and gender. Imprecision: moderate size of the pool of original articles (n = 6). There was no statistical analysis applied. Indirectness: The review article investigated global regions. Imprecision: a small pool of research (n = 1).

d Risk of Bias: There were reported only self-reports. Indirectness: The review article investigated global regions. Imprecision: There was a small pool of research (n = 2).

e Risk of Bias: One original article was extracted. Indirectness: The review article investigated global regions. Imprecision: a small pool of research (n = 1).

f Risk of Bias: Original articles were extracted. Indirectness: The review article investigated global regions. Imprecision: There was a small pool of research (n = 3).

g Risk of Bias: Original articles were extracted. Indirectness: The review article investigated global regions. Imprecision: a small pool of research (n = 1).

h Risk of Bias: There were reported only qualitative data. Grey literature was considered in fishing communities.

i Risk of Bias: One original article was extracted. Imprecision: There was a small pool of research (n = 1).

j Risk of Bias: Original articles were extracted. Imprecision: There was a small pool of research (n = 2).

k Indirectness: The review article investigated global regions.

l Indirectness: The review article investigated global regions. Imprecision: There was a small pool of research (n = 2).

m Indirectness: The review article investigated global regions. Imprecision: There was a small pool of research (n = 3).

### 3.3 Articles' findings

The findings were synthesized according to the four socio-economic statuses of the Western Pacific Region and were further categorized in the countries of that region. Original articles were extracted when needed to offer a more detailed description of the findings and to facilitate the findings’ synthesis. Note that the latter process was followed frequently due to limited information about mental health outcomes in the studied regions.

#### 3.3.1 High-income countries

##### 3.3.1.1 Australia

One article focused on the population of Australia [65], and three studied Australia among other regions [52,66,67]. Most articles focused on droughts and droughts’ influence on rural populations.

Droughts associate with a decline in the economic and mental well-being of farmers

[65] reported an increased suicide rate among male farmers and its possible influence due to droughts. To understand this better, we extracted the findings of three original studies [48,49,69]. [49] studied the influence of a prolonged drought in Victorian (2001–2007) on the suicide rate among farmers. Suicides were accessed via the Victoria Institute of Forensic Medicine. The findings indicated that suicide rates in farmers accounted for 3.1 % (n = 110 in total), of the total suicide rates in the Victorian population (n = 3523) during the drought period. However, male farmers accounted for 95 % of the reported farmer suicides, suggesting that male farmers had a higher risk of suicide during droughts. When looking at the age groups of the110 farmers’ suicides, middle-aged to older adults (30–59 years old) accounted for 63 % (n_30–39years_ = 22, n_40–49years_ = 24 _and_ n_50–59years_ = 23), young adults (<30 years) accounted for 17 % (n = 19), and older adults (≥60 years) accounted for 20 % (n_60–69years_ = 10, n_70–79years_ = 4 and n_80+ years_ = 8) of suicides during the drought period [49]. The latter suggests that middle-aged to older farmers might be at higher risk of suicide than younger ones. Nevertheless, these observations are based only on descriptive analysis of suicide percentages, and they lack evaluation of the findings using statistical tests and investigation of covariates (e.g., pre-existing mental health conditions). [48] provided a qualitative approach to explain the possible link between droughts and suicide rates in male farmers. These factors included farmers increased financial concerns when seeing their business failing, being unable to provide an income to support their family, having decreased self-worth, and being hesitant to seek help [48].

Extending Alston's [48] qualitative findings, [69] provided a quantitative approach to measuring psychological distress and coping style in 309 drought-affected farmers. Psychological distress was measured using the Kessler Psychological Distress Scale, and coping style was measured using the 15 subscales of the COPE inventory. The findings identified no significant differences between male and female farmers' psychological distress. However, age groups showed specific patterns of psychological distress. Specifically, younger farmers (25–54 years) did not show significant differences from older farmers (65–74 years), but they did show significantly higher distress from the middle-aged group (55–64 years). In addition, the multiple regression analysis showed that mental and behavioural disengagement, venting, suppression of completing activities, and alcohol/drug use were significant positive predictors of psychological distress. Finally, it is worth noting that the most commonly reported coping styles in response to drought were acceptance, active coping, and planning. Conversely, religion, denial, alcohol/drug, and behavioural disengagement were the least common coping styles [69].

A more recent systematic review reported additional findings about the influence of Australian droughts on farmers [52]. The authors reported farmers' increased concerns about job security and decreased income during drought periods (e.g., due to limited water supply; [52,70]). In addition, increased emotional challenges (e.g., increased solastalgia; a term that describes distress caused by environmental changes in the homeland) during droughts were linked to the loss of green land and landscape changes that influenced farmers' sense of home in farming families [52,71]. Finally, younger farmers (<35 years) who lived on their farms and had increased financial burdens had higher drought-related stress in the farming population [51,52]. Another study showed that the duration of drought significantly increased drought-related distress (measured by Kessler Psychological Distress Index) in young rural women but not men, while there was not a statistically significant difference in distress between farmers and non-farmers [50,52]. However, the authors reported that rural women were overrepresented in their study, and thus, findings must be interpreted with caution.

Droughts associate with a decline in economic, mental, social, and community well-being in indigenous populations

Aboriginal Australian communities are reported to be at risk of declining well-being during droughts [66] reported one qualitative study that investigated the influence of prolonged droughts on the social and emotional well-being of the rural Aboriginal communities in the New South Wales area [72]. We extracted the findings of the original article to understand this better. [72] reported that prolonged droughts negatively impacted Aboriginals' cultural identity and social connectedness, especially in the elderly. For example, drought caused the loss of rivers that were once social gathering spaces. Some individuals reported behaviors that disgraced their culture and disrupted the community's cohesion, such as alcohol use and increased aggression between community members. Displacement from their homeland due to droughts was also associated with a loss of the sense of home, unemployment, increased nostalgia for their homeland, and loss of their sense of community. The decline in their social well-being was followed by a decline in their homeland's economic development and local services, including health services [72].

Floods associate with increased substances use

[67] reported a small pool of research on how a flood affected Australian populations. An increase in self-reported alcohol, tobacco, and medication use was found among the flood victims. Finally, [67] reported one study that found no flood-related increase in depression symptoms of older Australian adults and another study that reported no increase in suicide rates in response to floods.

##### 3.3.1.2 Republic of Korea

Typhoons associate with PTSD symptoms

[68] reported one study about the influence of 2002 Typhoon Rusa on primary school students [73]. We extracted the original article to understand the findings better. The authors used the Post-traumatic Stress Disorder Reaction Index for Children (PTSD-RI) to assess PTSD-related symptoms. The findings showed that 67 % of students reported fear of typhoon recurrence, and 13 %–17.2 % reported sleep disturbance, difficulty in concentration, and guilt. When assessing the accumulation of PTSD symptoms, 12.3 % showed moderate or severe PTSD symptoms, and 22.7 % had mild PTSD symptoms [73].

#### 3.3.2 Upper middle-income countries of continental Asia

There were no review articles focusing on the continental Asia regions. However, there were limited referrals about the Philippines and Vietnam in articles that studied more broad regions [36,44,68]. Thus, below, we outline the findings from this small pool of research that studied associations between typhoons and mental health outcomes. The findings identified that typhoons were associated with PTSD and depression in the Philippines and Vietnam.

##### 3.3.2.1 Philippines

Typhoons associate with depression, acute stress, and PTSD symptoms. Three individual differences: trauma centrality, positive metacognition, and coping style play a crucial role in mental health outcomes

[44] reported a small section in their review article with two studies focusing on the influence of typhoons on victims' mental health. We extracted the findings of the two original articles to understand this better. [74] studied children and adolescent victims of Typhoon Washi. They measured acute stress using the Acute Stress Disorder Interview based on the Diagnostic and Statistics Manual IV (DSM-IV) and depression based on the Depression Self-Rating Scale for Children. The findings showed that the centrality of the traumatic event (an indicator of how much the traumatic event dominates victims' life experiences and plans) was positively associated with acute stress symptoms and depression. Interestingly, the intensity of the symptoms increased with more vivid traumatic memory. However, the authors did not measure the prevalence of acute stress and depression in the victims [74].

[75] studied the mental health of student victims 3-months after Typhoon Haiyan. They focused on PTSD symptoms measured by the Post-traumatic Cognitions Inventory and found a 16.14 % prevalence of PTSD symptoms. PTSD symptoms were also associated with victims' positive metacognition and emotion coping style (a) extinguishing: "confidence in extinguishing perseverative thoughts and emotions" (b) setting: "confidence in setting flexible and attainable hierarchies of goals". Specifically, the findings showed high scores in "extinguishing" and "setting" associated with decreased PTSD symptoms and a more positive coping style in victims' lives, such as increased appreciation of life and personal growth [75].

[68] reported some of the above studies by also mentioning difficulties in identifying and treating mental illness in the Typhoon Haiyan victims because of mental health stigma.

##### 3.3.2.2 Vietnam

Typhoons associate with increased depression and panic disorder symptoms

[36] studied the association between extreme weather events and mental health in developing countries, including Vietnam. The authors reported one study that studied mental health outcomes 3-month post the 2006 Typhoon Xangsane [76]. Specifically, the authors [76] measured PTSD via the National Women's Study PTSD module, Major depressive disorder, Panic disorder, and general anxiety via the Structured Clinical Interview for DSM-IV. The findings showed a 2.6 %, 5.9 %, 9.3 %, and 2.2 % prevalence of PTSD, major depressive disorder, panic disorder, and general anxiety disorder, respectively [76].

#### 3.3.3 Low to upper-middle-income countries of Pacific Island states

Two recent review articles (published in 2022) studied Pacific Island states using only qualitative studies. One of the review articles focused on Pacific Islands, broadly reporting the psychological and cultural experiences of Islanders in response to climate-related dislocation [53], and one review article studied the general health impacts of climate change in Micronesian fishing communities [58]. Overall, findings indicated a decline in multiple well-being aspects (economic, mental, social, and community well-being) in fishing communities and the dislocated populations, but also coping mechanisms and strategies to prevent the decline in well-being. We summarize below Hodgson et al. [58] and Yates et al. [53] findings. There was only one case [77] in which we extracted the findings from the original article to facilitate a better understanding.

Climate-related dislocation associated with a decline in economic, mental, social, and community well-being

[53] reported that Pacific Islanders face a high probability of dislocation from their homeland due to climatic events such as sea level rise, king tides, and receding coastline influenced by climate change (e.g., global warming). In addition, the authors identified that cross-border and internal mobility populations might face similar and also different challenges [53].

Islanders that left their ancestral lands to migrate cross-border (e.g., to Fiji, New Zealand, Australia, U.S.) faced an accumulation of displacement stressors [53]. The listed stressors include high unemployment, work exploitation, and non-transferable educational credits in the migrated location. In addition, migrants faced the inability to access healthcare services, racism, and language-related barriers in the relocated area. Nostalgia was also reported when remembering their homeland and sadness, despair, and fear about the members that remained behind. Interestingly, Tuvaluans that migrated reported greater concern for their loved ones in Tuvalu than the Tuvaluans who remained in their homeland. Finally, when focusing on the Kiribati population, the dislocation challenges seemed to impact more women than men due to their limited experience with overseas traveling. [53] also reported that the above stressors are associated with depression, anxiety, and stress in the Pacific Islands literature of dislocated populations, implying a possible increase in clinical mental health symptoms. However, the latter remains speculation as there were no studies investigating this [53].

Community cohesion also showed a decline that was associated with increased negative emotions among the members of the migrated populations [53]. For instance, some dislocated migrants living in New Zealand reported sadness because of the reduced participation in communal gatherings and cultural events compared to their past experiences in their homeland. These findings were extended by increased fear about losing cultural identities such as mother language and ancestral traditions. Adaptation of dislocated populations to an individualistic culture was suggested as a factor that might negatively influence their practices based on collective culture (e.g., Tuvaluans) [53].

Internal migration was also reported to influence community cohesion and community-shared fear due to climate change [53]. For example, dislocated Solomon Islanders reported reduced participation in religious and cultural gatherings. Part or whole communities that moved reported conflicts with other communities that live in their relocation areas, such as disagreement over land and reduced sea rights for farmers and fishers. Fishers also reported traveling a long distance from their relocated area to their job's fishing location. Finally, communities reported frustration about living in an unfamiliar land, facing climate-related events they did not cause, and the passivity of the "bigger countries" accountable for climate change [53,77].

Coping mechanisms and protective factors in dislocated populations

Despite the dislocation challenges, coping mechanisms and beneficial practices were identified in internal and cross-border dislocated populations [53]. Cultural exchange gatherings (e.g., singing and sharing migration stories) between different islander populations increased their sense of social support in the relocated area, highlighting the importance of active community organizations. In addition, cross-border migrants' solidarity with indigenous populations of the relocated area increased their sense of belonging. Internal migrants reported a preference for whole-village relocations to help maintain their identity. In addition, the ability to choose the relocation site and produce a similar town planning (e.g., home's building materials) to the previous one was suggested as a beneficial factor for a smooth transition between the past and the new living conditions and to decrease the chances for community disruption. For instance, the dislocated villagers in Vanuatu were satisfied with their new living conditions when they planned their relocation according to their needs. In contrast, the relocation of a Fiji villagers' community to a hilly area prevented the elderly from participating in community activities due to access issues considering their mobile condition. Interestingly, remembrance of the dislocation history of ancestral communities was identified as a factor that could help decrease dislocation fear [53].

Extreme weather events, increased temperatures, and changes in the marine ecosystem are associated with a decline in economic, mental, social, and community well-being in subsistence fishing communities

Hodgson et al. [58] comment on climatic factors that could lead to the forced migration of subsistence fishing communities of the Pacific Islands. Specifically, the authors identified that increased temperatures, storms, and changes in coastal marine resources could create survival problems related to employment and nutrition (e.g., food insecurity in Kiribati fishers due to limited reef fish). In addition, food-related insecurity was reported to disproportionally influence women who tend to prioritise the survival of their family members and risk their own nutrition [58].

Hodgson et al. [58] also reported a decline in individual and community well-being in response to climate change in fishing communities. Climate change was associated with reports of stress, anger, depression, sleep disruption, and a sedative lifestyle. Outdoor activities that could offer enjoyment and opportunities for physical activity, social gathering, and community bonding were at risk due to increased heat. Women and elderly populations were reported among the most vulnerable populations suffering from the above climate-related effects [58].

Coping mechanisms and protective factors in fishing communities

Local and global strategies were proposed to decrease climate-related stressors in fishing communities by Hodgson et al. [58]. For example, local services could offer educational programs to tackle food insecurity and unemployment, such as practices focusing on nearshore pelagic fishing rather than reef fishing. In addition, counseling services, such as Tuvalu's Skills for Life Adjustment and Resilience program, could provide mental health support and coping mechanisms tailored to climate change-related stressors. Finally, in line with the observation of [53] about migrated populations, community-led relocation was recommended.[58]

The authors also suggested future research about the practices of post-harvest fish preservation that could offer food security and opportunities for physical activities due to the reported decline in outdoor fishing activities [58].

#### 3.3.4 Low to upper-middle-income countries of continental Asia

China

One review article focused on the population of China [59], and four studied China among other regions [36,44,67,68]. However, none of the review articles investigated mental health changes in response to climate change in the Chinese population but reported general health outcomes, including mental health. Thus, original articles were extracted if needed to understand our findings better. Overall, the most studied climatic event that influenced mental health was flooding. The most frequently reported mental health outcome in response to any of the studied climatic events was Post-Traumatic Stress Disorder (PTSD). We outline below in more detail our findings about the influence of the different climatic events on the mental health of the Chinese population.

Extreme weather events associate with post-traumatic stress disorder (PTSD), anxiety, and depressive symptoms
Floods

[59] studied the influence of extreme temperatures, rainfalls, sea level rise, and extreme weather events on general health outcomes, including mental health. The authors reported that mental health was an understudied topic among the selected studies and was associated only with two extreme weather events; floods and typhoons. Floods were the most studied extreme weather event focused on the 1988 flooding of Dongting Lake in central China. Specifically, populations that suffered from the 1988 flood reported a ∼ 10 % prevalence of Post-Traumatic Stress Disorder (PTSD, measured using the DSM-IV). Longitudinal studies suggested an up to 20 % prolonged prevalence of PTSD (measured using the PTSD Checklist-Civilian version or DSM-IV) in flood victims, with the most recent estimation being 9.5 %. Risk factors for PTSD in the flood victims were the female gender, elderly, and flood characteristics (type and severity). In addition, stressful events related to the flood (e.g., losing a loved one) and negative coping styles, such as using intoxicating substances, increased the likelihood of a prolonged PTSD diagnosis [59,78]. In contrast, social support was a protective factor against the flood victims' short- or long-term PTSD. One of the original studies also reported a 9.4 % anxiety prevalence (anxiety was measured using the Zung Self-Rating Anxiety Scale) and 6.2 % PTSD (PTSD was measured using the PTSD Checklist-Civilian version) and anxiety prevalence in the flood victims. Of those with PTSD, 64.5 % also suffered from anxiety [59,79]. Anxiety risk factors were similar to PTSD; female gender, experiencing flood-stressor-related events, emotional instability, and low social support [59,79]. Four more review articles focused on the 1988 flooding of Dongting Lake [36,44,67,68] and mentioned overlapping studies with that of [59].

##### Typhoons

Typhoons were also linked with PTSD [59]. The two original articles [80,81] reported in the review article of [59] were extracted to look more closely at their findings. [80] investigated the effects of Typhoon Morakot on PTSD (measured with the Mini-International Neuropsychiatric Interview for Children and Adolescents) in adolescent victims. Their findings showed a 25.8 % prevalence of PTSD. Risk factors for PTSD diagnosis in the victims were the female gender, previous PTSD diagnosis related to traumatic events, physical injury, and loss or injury of family members during the typhoon [59,80]. Finally [81], investigated the influence of the Rammasun typhoon on PTSD in the Hainan Province victims with clinical PTSD diagnosis. Specifically, they studied the association of PTSD with platelet serotonin levels (5-HT, a biomarker linked to PTSD symptoms in some literature [82]) between victims and non-victims (control group). Their findings showed lowered platelet 5-HT among people living with PTSD compared to a control group. However, as the author's primary aim was to associate PTSD with platelet 5-HT, they did not measure PTSD prevalence among the victims [59,81].

##### Tornados

Sharpe & Davison [44] reported 11 studies in their scoping review that focused on the 2016 tornado in Yancheng City and its influence on adolescent victims. The majority of studies focused on PTSD. One study reported a three-month 57.5 % and 58.7 % prevalence of PTSD and depression, respectively, in middle school students after the tornado. Students who reported injured family members due to the tornado were at higher risk for PTSD and depression [44].

##### Storms

Sharpe & Davison [44] reported a high risk of PTSD (measured with DSM-V, Child PTSD Symptom scale, or Impact of Event Scale-Revised) after rainstorms and snowstorms in middle school students based on three studies. Specifically, there was a 15.2 % PTSD prevalence one week after rainstorms. PTSD was linked positively with walking distance during the snowstorm in the Hunan province of China in January 2008 [44].

## 4 Discussion

### 4.1 Summary

The present scoping umbrella review collected review articles that followed a screening selection process against eligibility criteria to identify the relationship between climate change exposures and mental health in the World Health Organisation Western Pacific Region. Climate change exposures of interest included any climate change-related event. Mental health outcomes of interest included 1) non-clinical symptoms: decline in mental, social, economic, and community well-being and 2) clinical symptoms: increase in Post-Traumatic Stress Disorder (PTSD), depression, anxiety, and acute stress symptoms. Ten articles were eligible after screening (see Table 2). Most articles investigated global regions (n = 5) and focused exclusively on mental health outcomes (n = 7). Interestingly, the most frequently reported mental health outcomes, irrespective of the climatic exposure, were a decline in mental well-being and an increase in PTSD symptoms. It is also essential to note that the quality assessment of the observed relationships between a given climatic exposure and a mental health outcome was dominated by low or very low-quality assessments. The main reason for the latter was the limited number of articles for a given mental health outcome, highlighting the need for further mental health research in WPR. This is not surprising considering that there is scarce research on climate change and mental health globally, which is reported as a major knowledge gap in the climate change literature [83].

It is also pivotal to highlight that the primary aim of our study is to provide an overview of the present literature and guide further research in specific geographical regions within WPR. However, the presented findings are not intended for direct inter-regional comparisons regarding the variance or intensity of mental health outcomes. Additionally, the focus on particular mental health outcomes within a region is dictated by the findings from the review articles incorporated in our research. This does not preclude the significance of other mental health outcomes not highlighted herein. The researchers’ choice to study these mental health outcomes could stem from various factors, such as existing literature, prevailing research priorities, available funding, and contemporaneous research opportunities.

To facilitate a comprehensive grasp of the results and possible biases, our findings are summarised below and further reported in relation to the global literature discussing the interplay between climate change and mental health.

**Key findings**

The findings identified that each region faces a different combination of climate change-related events and has differential needs depending on populations' employment type and cultural links to the land. Finally, demographic characteristics showed mixed patterns depending on population, culture, employment, and climatic exposure. In more detail.

- **Region:** Prolonged droughts were linked to a decline in rural Australian populations' economic and mental well-being [52,65,66], while research about the influence of droughts on suicide rates among Australian farmers was inconclusive. Pacific Islanders reported increased well-being stressors related to forced migration from their homeland and employment threats due to sea level rise and marine ecosystem changes [53,58]. Chinese populations faced various climatic events (floods, typhoons, tornados, storms) associated primarily with PTSD symptoms [36,44,59,67]. Similarly, populations in the Republic of Korea, the Philippines, and Vietnam were associated with increased PTSD symptoms when facing typhoons [36,44,68].
- **Employment:** Farmers and fishers were identified as vulnerable populations facing an accumulation of climate change-related stressors and increased risk of a decline in their communities' well-being [52,58,65,66].
- **Culture:** Indigenous people such as Aboriginals in Australia and Pacific Islanders with increased cultural tights to land were identified as high-risk populations facing increased climate change-related stressors and increased risk of declining their communities' well-being [52,53,58,65,66].
- **Demographic risk factors:** Gender and age seemed to regulate the relationship between climatic exposures and mental health outcomes [52,53,58,65,67]. However, their influence was not unified or conclusive across regions and climatic exposures.
- **Protective factors:** social support from local services and communities and having an active role in the decision-making plan for implementing life changes due to climate change (e.g., migrated location) were associated with positive views and mental health outcomes from the studied populations [53,58,59].

### 4.2 Key findings in relation to the broader literature

Prior climate change literature highlighted that a country's socio-economic status plays a crucial role in how their populations will receive climate change influences [36,43,44]. Thus, in this section, we separate our findings according to the socio-economic status of a given region/country. We also provide future recommendations for research and potential policy directions in a given country in the WPR, considering the current knowledge and gaps. It is essential to clarify that our recommendations aim to enhance academic discussion and are not intended to critique or diminish policymakers' efforts in these areas. Please also note that the Republic of Korea, Philippines, and Vietnam are not further discussed here due to limited findings with low-quality assessment in our review. Instead, the findings of Australia, the Pacific Islands (broadly), and China are discussed.

#### 4.2.1 High-income countries: Australia

Farmers and aboriginals are at risk of declined mental health during droughts

Droughts were the most studied climate change-related event identified in our review for Australia. This is not surprising since prior literature indicates droughts as a major concern for the Australian economy, specifically its agricultural production [84]. For instance, a 1 % fall in Australian gross domestic product (GDP) growth was reported due to a decrease in agricultural products during the prolonged drought between 2002 and 2003 [84]. In addition, many studies report rural populations, specifically farmers, at high risk of declining mental health due to droughts [[48], [49], [50], [51], [52]]. Australian rural populations are reported with decreased access to mental health services [85,86], increased helplessness, psychological distress [87], and suicide rates [65] compared to urban populations, which could be partly explained by climatic challenges in addition to their socio-economic status [84]. Among the rural Australian populations, farmers are identified as at high risk of developing mental health issues [65,86]. In line with the above reports, our findings showed consistent reports of Australian farmers' increased financial concerns and mental well-being-related stressors during drought.

Interestingly, our findings also showed that while farmers faced an accumulation of psychological stressors, they did not show a higher suicide rate or distress when compared to non-farmer rural individuals [52]. Berry et al. [84] offered a possible explanation. It is likely that farmers and rural populations, more broadly, show increased resilience due to their support from local communities and charitable organizations that are active in rural areas [84]. It is also observed that during disasters, social cohesion increases, such as strengthening rural identity and positively influencing individuals' mental health [88]. However, it is essential to note that local rural communities also face threats due to climate change, such as the displacement of younger generations due to failing family businesses and poverty, which may weaken the cohesion and support they can offer [84].

The influence of demographic differences on the relationship between droughts and farmers' mental health outcomes was mixed in our findings. For example, when investigating suicide rates, male farmers seemed at higher risk [49,65], but when investigating psychological distress, findings were not significant regarding gender differences [65,69]. In addition, some studies identified older and younger populations at risk while others only one of these two age categories [65]. Our findings also support that other covariates, in addition to demographic characteristics, might also explain the mixed findings. For example, coping styles may influence mental health outcomes [69]. Thus, prediction models that account for covariates could give a clearer picture of which individual characteristics position farmers' mental health at risk during drought.

Aboriginal populations emerged as another high-risk population for declining mental health during drought. Specifically, aboriginals showed a decline in all studied well-being measures; economic, mental, social, and community well-being. Our findings align with broader observations of the climate change literature that identify Aboriginals as a high-risk population to suffer from climate change [[89], [90], [91]]. For instance, a recent article compared the climate-related exposures in Aboriginal compared to non-Aboriginal populations in the New South Wales (NSW) of Australia [89]. The findings showed that 26 % of NSW Aboriginal populations lived in areas with n > 7 heatwave days annually, 13 % faced moderate to high rainfall, and 32 % lived in areas affected by prolonged droughts compared with 9 %, 3 %, 7 % of non-Aboriginal populations respectively [89].

Other climate change-related exposures that may influence mental health

It is essential to acknowledge that Australia's climate is highly variable and thus that the impacts of climate change are not limited to droughts [92,93]. In some areas of Australia, we can expect an increase in the frequency and intensity of other climate-related events, such as sea surface temperatures, rainfall, and floods [92,94]. In our findings, floods were associated with increased substance use (e.g., tobacco), but please note that this observation comes from a small pool of research.

Summary

In Australia, droughts exert a pronounced strain on rural communities. Among these, farmers emerge as a group particularly vulnerable to escalating mental health concerns. Yet, a noteworthy observation is that despite these pressures, the suicide rates among farmers have not surged noticeably compared to other rural groups. This resilience, potentially rooted in community cohesion, is being challenged by the migration patterns of younger individuals, driven predominantly by economic factors. In parallel, Aboriginal communities face profound setbacks during droughts, marked by economic, mental, and social declines. Given Australia's shifting climate—manifested in changing rainfall trends and more frequent floods—an in-depth investigation into the attendant mental health implications is crucial. These findings underscore the urgency for expansive studies on the mental health ramifications stemming from various climatic phenomena, including but not limited to heatwaves.

Policy considerations

Considering the summary of our findings, enhancing rural mental health support—tailored for farmers—could be beneficial. Emphasizing community integration and retaining younger demographics may help sustain community unity. Culturally attuned mental health initiatives, co-developed with community leaders, could be effective for Aboriginal communities. Policy frameworks for the broader agricultural community could contemplate financial cushions like subsidies, loans, and drought-centric insurance to mitigate drought stress.

Future research directions

A recent scoping review on global patterns about climate change and mental health identified Australia with the most research articles (n = 34) [11]. Adding to this, Daghagh Yazd et al. [52] suggest more thorough research on the influence of climate change on farmers' mental health. Thus, we recommend future research to conduct systematic reviews specific to the Australian population to understand their needs better. Our findings also suggest research gaps that encourage more primary research. For instance, there is a need to investigate the resilience observed in Australian farming communities to understand the factors behind this and inform strategies that could benefit wider communities. In addition, although there is a pool of research investigating the relationships between high temperatures or heat waves and mental health, a more in-depth understanding of that relationship is identified as a gap in climate change research [95]. Finally, future research could further investigate the association between floods or rainfall and mental health outcomes.

#### 4.2.2 Low to upper-middle-income countries of Pacific Island states

Pacific Islanders face climate related-migration

Pacific Islanders face immediate climate change threats, such as losing coastal territories due to rising sea levels and, consequently, forced migration [53]. Our qualitative findings majorly reported that internal and cross-border migration of Pacific Islanders was associated with a decline in all studied well-being measures. However, there were no reports of longitudinal mental health outcomes or quantitative measurement of well-being and clinical symptoms.

Several knowledge gaps require a more thorough investigation of the relationship between climate change and mental health in the Pacific Islands. For instance, the Pacific Islanders have among the highest rates of suicide in the Western Pacific region [96]. However, climate change's influence on this observation is to be investigated. In addition, Pacific Islanders face climate-related disasters such as tropical cyclones, floods, and droughts, with emerging research indicating the negative physical health consequences [97]. However, our findings did not report any relationships between the influence of a specific climatic event and mental health.

Access to health services could be critical in preventing or mitigating the adverse mental health outcomes that arise from climate change stressors. However, Pacific Islands' mental health and post-disaster services are considered poor [98]. The reasons are limited infrastructure, funding, and the small number of mental health professionals in the Pacific Islands [98]. To overcome these challenges, Dawes et al. [98] propose de-centralizing the Pacific mental health services and introducing programs to support vulnerable groups such as children that will increase mental health awareness in Pacific Island communities. Finally, programs that can help Pacific Islanders develop skills to adapt to new climatic conditions, such as Tuvalu's Skills for Life Adjustment and Resilience program [58], could also have positive mental health outcomes.

When trying to understand the needs of Pacific Islanders and policies that could help increase their resilience to climate-related hazards, it is also essential to understand their unique culture. Pacific Islanders report a strong ancestral connection to their land, and their cultural identity is linked to religious and cultural traditions and participatory community activities [53,97,99]. In our findings, selecting the relocated destination was a beneficial strategy for a smooth transition between the past and the relocated area that allowed the continuation of social activities and traditional practices. In addition, literature reports that Pacific Islanders are highly resilient populations, possibly because of their strong community links [99].

Summary

Pacific Islanders face pressing climate-related challenges, primarily due to escalating sea levels that compel forced migrations, leading to subsequent declines in well-being. A discernible gap persists in longitudinal studies delving into such environmental shifts' long-term mental health effects. Notably, the region, which registers some of the highest suicide rates in the Western Pacific, has yet to extensively investigate the potential role of climate change in this phenomenon. Additionally, the nuanced mental health implications of specific climatic events are yet to be comprehensively examined. Demographics such as fishing communities emerge as especially vulnerable in this context. When crafting policies for Pacific Islanders, it is crucial to recognize and respect their profound cultural identity, deeply anchored in ancestral ties and communal values.

Policy considerations

Addressing the intertwined challenges of climate change and mental health in the Pacific Islands might benefit from several considerations. One potential avenue could be to explore policies that facilitate smooth transitions for Pacific Islanders who face displacement due to rising sea levels. This involves looking at physical infrastructure and ways to preserve and continue their traditional activities. Another suggestion might be to examine the current state of the Pacific Islands' mental health infrastructure. Embracing a decentralized service model might offer a more localized, community-centric approach, and perhaps, by channeling resources into training and incentivizing professionals, the region's mental health framework could be strengthened. For those communities particularly vulnerable to the effects of climate change, like the fishing communities, it might be worthwhile to consider targeted interventions. Supporting their transition towards more sustainable practices or introducing alternate livelihoods could serve as a cushion against adverse mental health impacts. Lastly, drawing inspiration from successful adaptation programs, such as Tuvalu's Skills for Life Adjustment and Resilience, might offer a blueprint for designing initiatives that equip communities to navigate climate change's multifaceted challenges. These suggestions, viewed collectively, could potentially offer a comprehensive, culturally-sensitive approach to fostering the well-being of Pacific Islanders in an evolving environmental context.

Future research directions

Qualitative studies provided valuable insights into the influence of climate change on well-being. Nonetheless, in line with Dawes et al. [98], we recommend conducting quantitative studies to reach a holistic understanding of the Pacific's post-climatic disaster mental health outcomes, including clinical symptoms assessment. Our findings also highlight the need to explore the potential role of climate change in the reported high suicide rates among Pacific Islanders. In addition, given the limited data available, investigating the impact of specific climatic events on mental health of Pacific Islanders is essential. Finally, we further recommend a more in-depth research about the influence of climate change on community well-being and ways to prevent its decline, especially in the migrated Pacific Island populations.

#### 4.2.3 Low to upper-middle-income countries of continental Asia: China

Climate change-related events pose mental-wellbeing risks for young populations

China experiences various climate change-related events across different regions [100]. In our review, floods were the most studied climatic exposure, but findings were also reported for typhoons, tornados, and storms (rainstorms and snowstorms), with a focus on children and adolescents. The studies primarily investigated clinical symptoms of PTSD, anxiety, and depression, with PTSD emerging as the most frequently studied mental health outcome.

The young population, especially children, is reported among the most vulnerable populations globally, with increased psychological effects when facing climate change challenges [4,6,9,101]. These psychological effects include PTSD, anxiety, depression, phobia, sleep disturbances, attachment disorders, and negative coping styles like substance abuse [101]. Furthermore, mental health consequences for young people could be devastating due to their rapidly developing brains and, thus, their vulnerability to developmental mental health issues when facing traumatic events [4]. However, less is known about the indirect effects (e.g., climate anxiety) of climate change on young Chinese or global populations, while the literature suggests increased concerns over climate change compared to any other age group [4,101]. Finally, vulnerable populations, such as the elderly Chinese, have also been identified [102].

Other climatic events or events related to anthropogenic interventions are reported in China. These include haze [103], loss of green spaces [104], seasonal temperature variations [105], and precipitation [106]. However, we did not identify review articles investigating mental health outcomes relevant to these events.

Summary

China faces diverse climate challenges, with floods being the most studied. Young populations, particularly children, appear vulnerable, showing increased psychological effects from climate events, including PTSD, anxiety, and depression. The indirect impacts of climate change on youths, such as climate anxiety, remain underexplored. Meanwhile, other climatic events and anthropogenic interventions like haze and loss of green spaces are acknowledged but lack comprehensive mental health research.

Policy recommendations

Considering the complexities posed by climate change in China, it might be beneficial to give attention to the mental well-being of the nation's youth when contemplating potential policies. A possible avenue could be emphasizing the mental health of young individuals during disaster preparedness phases. This might entail considering the development of specialized interventions post-climatic events to address potential trauma manifestations, such as PTSD, anxiety, and depression. Beyond direct impacts, it could be worthwhile to consider climate change's more nuanced, indirect psychological effects, like climate anxiety. In this context, introducing psycho-educational programs might be valuable in fostering resilience within this group. As other environmental concerns, such as haze or the loss of green spaces, increasingly influence urban areas, policy frameworks could reflect these particular challenges.

Future research directions

Future research could investigate the indirect effects of climate change on young Chinese populations, guiding policy interventions. In addition, review articles could synthesize existing findings about haze, green space, seasonal temperature variations, precipitation, and mental health outcomes to provide a clearer understanding of the topic and its gaps in China. Such reviews could offer an a better understanding of which individual differences are risk factors during specific climatic exposures.

#### 4.2.4 Key research gaps & future directions

In our umbrella scoping review, several issues were identified in the climate change and mental health literature in WPR. Interestingly some of our observations (see points 2, 4–6 in Table 4) are also reported in recent reviews evaluating methodological issues and research gaps in the global climate change literature [107,108].

**Table 4:** Key research gaps and future research directions about the influence of climate change on mental health in the World Health Organisation Western Pacific Region (WHO WPR).

Gaps	Future directions
A limited number of review articles were identified for a given country of WPR (n = 5), and an overall low-quality assessment of our findings regarding climatic exposures and mental health.	Conduct systematic reviews specific to a given country of WPR, gathering information from research articles to understand better the relationship between climate change and mental health for that country.
Communities seem essential for Pacific Islanders, farmers, fishers, and Australian Aboriginals. However, there was limited research on climate change's influence on these communities, specifically the communities' well-being.	More in-depth research about how communities are impacted by climate change could help design policies on keeping them active.
Pacific islanders seemed to be the population that faced the most immediate threats of forced migration. However, there was limited research on policies that could ensure a smooth integration into the relocated destination while maintaining their cultural identity.	Further investigation into internal and cross-border migration needs could help design policies allowing Islanders to integrate into their relocated area while keeping their cultural identity.
Longitudinal studies were majorly reported for the assessment of PTSD symptoms. However, there was limited research on the long-term mental health effects of general well-being.	Conduct studies assessing short- and long-term well-being, specifically in regions that reported only clinical symptoms (e.g., China).
The possible confounding role (moderating or mediating) of individual differences in the relationship between climate change and mental health was not systematically investigated.	Future studies could focus on building multivariate prediction models investigating the influence of climate change and mental health outcomes to understand better the risk and protective factors against a decline in mental health.
There was a heterogeneity of self-reported measures used to evaluate clinical symptoms.	Future review articles could acknowledge the mental health measurement methods in their findings section to allow space for criticism and avoid misjudgment of non-clinical conditions as clinical.
Qualitative studies primarily reported on the evaluation of well-being.	Quantitative studies of well-being aspects (economic, mental, social, and community) could strengthen the confidence of the observed patterns in a given WPR country.
Sleep disturbances were rarely addressed in the review articles. We consider this a major knowledge gap since sleep disturbances commonly co-occur with non-clinical and clinical mental health symptoms. Moreover, a bidirectional relationship between sleep and mental health is suggested (sleep could be both a symptom and cause of mental health outcomes) [37,38,109].	Future studies could focus on investigating the influence of climate change on mental health and sleep disturbances (e.g., sleep duration, sleep latency, and the number of waking times).

#### 4.2.5 Limitations

Our scoping umbrella review has certain limitations. First, we included only review articles that followed a screening selection process against eligibility criteria to exclude possible misinterpretation or misjudgment of the literature. However, following this process, we may have missed some indications from grey literature that could strengthen or clarify our findings. In addition, we are missing primary research articles that studied the mental health consequences of climate change in the WPR that were not included in the ten selected review articles. We also excluded articles that investigated global regions and pooled their observations together, making it difficult to understand the findings for a given country/region in isolation. By doing this, we acknowledge that we may have missed some information. Finally, we included only English articles; thus, the information described in a different language was not assessed.

## 5 Conclusion

The limited global research about the influence of climate change on mental health was reflected in our scoping review that focused on the World Health Organization Western Pacific Region (WPR). However, emerging patterns identified a decline in mental well-being and an increase in PTSD symptoms as concerning. In addition, populations with close links to the land and their communities, such as Pacific Islanders, farmers, fishers, and Aboriginals, were identified as vulnerable populations during climate change challenges. Finally, considering the unique combination of climatic exposures and vulnerable populations in a given WPR country, we recommended in-depth research for each WPR country separately to guide climate change and mental health policies. Our suggestions included conducting systematic reviews for each WPRP country when there is the availability of research articles (e.g., Australia) and conducting quantitative mental health studies (e.g., in Pacific Islands). Finally, we reported that the lack of information about mental health and sleep disturbances in response to climate change is a major gap in climate change research of the WHO WPR.

## Funding statement

This work was funded by the World Health Organization under an agreement for the performance of work issued in behalf of the western Pacific regional office.

## Data availability statement

Data included in article/supplementary material/referenced in article.

## CRediT authorship contribution statement

**Aikaterini Vafeiadou:** Data curation, Formal analysis, Investigation, Project administration, Writing – original draft, Writing – review & editing. **Michael J. Banissy:** Conceptualization, Supervision. **Jasmine F.M. Banissy:** Data curation. **Julian P.T. Higgins:** Methodology, Resources. **Guy Howard:** Conceptualization, Funding acquisition, Supervision.

## Declaration of competing interest

The authors declare that they have no known competing financial interests or personal relationships that could have appeared to influence the work reported in this paper.
