# Supplementary material for: The influence of climate change on mental health in populations of the western Pacific region: An umbrella scoping review
Source: Heliyon. 2023 Nov 8;9(11):e21457. doi: 10.1016/j.heliyon.2023.e21457 (PMC10694052; doi:10.1016/j.heliyon.2023.e21457)
Supplement: Multimedia component 1 [file mmc1.docx]

# **Supplemental Materials**

## **Database search strings**

The database search strings were constructed by combining the climate change, mental health, and geographic keywords in one query or by synthesis of queries using "AND". The search was conducted on titles and abstracts (for CINAHL, PsycINFO, and PubMed), including authors' keywords when possible (for EMBASE, Scopus, and Web of Science). In addition, thematic terms were used according to the functionality of each database. For example, there were used Medical Subject Headings (MeSH; PubMed), descriptors terms (DE; PsycINFO), and explosion terms (/exp; EMBASE). In Table 1, we present the search string used in PubMed. The rest of the database searches were adapted based on the PubMed search string.

Table 1. The PubMed search string was constructed by synthesising three queries using "AND". In this table, the three queries are described separately.

| Keywords theme | Query |
| --- | --- |
| Climate Change | (("Climate Change"[Mesh] OR "climate change"[tw] OR "climate variability"[tw] OR "Global Warming"[Mesh] OR "Global Warming"[tw] OR "Greenhouse Effect"[Mesh] OR "greenhouse effect"[tw] OR "Climatic Processes"[Mesh] OR "Hot Temperature"[Mesh] OR "hot temperature*"[tw] OR "high temperature*"[tw] OR heat*[tw] OR drought[tw] OR "Climate"[Mesh] OR "Weather"[Mesh] OR weather[tw] OR precipitation[tw] OR rain*[tw]OR "humidity"[Mesh] OR humidity[tw] OR flood*[tw] OR storm*[tw] OR “sea level”[tw] OR “sea-level”[tw] OR glacia*[tw] OR melt*[tw] OR air quality[tw]) AND ((review[Filter] OR systematicreview[Filter] OR meta-analysis[Filter]) AND (english[Filter]))) |
| Mental Health | ("Mental Disorders"[Mesh] OR mental disorder*[tw] OR mental illness*[tw] OR "Mental Health"[Mesh] OR mental health[tw] OR mental disease*[tw] OR PTSD[tw] OR anxiety[tw] OR depression[tw] OR mood[tw] OR distress[tw] OR aggression[tw] OR worry[tw] OR suicide OR wellbeing[tw] OR well-being[tw] OR "social cohesion"[tw] OR "community well*"[tw] OR sleep[tw] AND ((meta-analysis[Filter] OR review[Filter] OR systematicreview[Filter]) AND (english[Filter]))) |
| West Pacific Region | (("western pacific"[tw] OR western-pacific region*[tw] or west pacific[tw] OR pacific[tw] or "Pacific Islands"[Mesh]) OR ("Asia"[Mesh] OR Asia[tw]) OR ("Australia"[Mesh] OR Australia[tw]) OR ("Brunei"[Mesh] OR Brunei[tw]) OR (Cook Islands[tw]) OR (French Polynesia[tw]) OR ("Guam"[Mesh] OR Guam[tw]) OR ("Hong Kong"[Mesh] OR Hong Kong[tw]) OR ( Macao[tw]) OR ("Mongolia"[Mesh] OR Mongolia[tw]) OR ("China"[Mesh] OR China[tw]) OR ("Japan"[Mesh] OR Japan[tw]) OR ("Laos"[Mesh] OR Laos[tw]) OR ("Cambodia"[Mesh] OR Cambodia[tw]) OR ("Singapore"[Mesh] OR Singapore[tw]) OR ("Malaysia"[Mesh] OR Malaysia[tw]) OR ("New Caledonia"[Mesh] OR New Caledonia[tw]) OR ("Philippines"[Mesh] OR Philippines[tw]) OR (Niue[tw]) OR ("Republic of Korea"[Mesh] OR Republic of Korea[tw]) OR (North Mariana Islands[tw]) OR (Pitcairn islands[tw]) OR (Tokelau[tw]) OR (Viet Nam[tw]OR Vietnam[tw]) OR (Wallis and Futuna[tw]) OR ("Papua New Guinea"[Mesh] OR Papua New Guinea [tw]) OR ("Samoa"[Mesh] OR Samoa[tw]) OR ("Tonga"[Mesh] OR Tonga[tw]) OR ("Marshall Islands" OR Marshall Islands[tw]) OR ("Micronesia"[Mesh] OR Micronesia[tw]) OR ("Nauru”[tw]) OR (Solomon Islands[tw]) OR ("Vanuatu"[Mesh] OR Vanuatu[tw]) OR (Kiribati[tw]) OR (Tuvalu[tw]) OR ("Fiji"[Mesh] OR Fiji[tw]) OR ("New Zealand"[Mesh] OR New Zealand[tw])) AND ((meta-analysis[Filter] OR review[Filter] OR systematicreview[Filter]) AND (english[Filter])) |

## Characteristics of the selected articles

Table 2. The ten selected articles' general characteristics and their studied regions in the Western Pacific Region are described here.

| General Characteristics | | | | Characteristics relevant to the Western Pacific Region (WPR)* | | |
| --- | --- | --- | --- | --- | --- | --- |
| Article | Number of primary studies | Studied Population | Studied region | Studied regions of WPR | Climate change exposures  associated with mental health outcomes | Mental Health Outcomes |
| 1 | 23 | Children up to 18 years old | Global (low- and middle-income countries; LMICS) | China  Philippines | China: 2016 tornado in Yancheng City, 1998 flooding of Dongting Lake, rainstorm disaster, snowstorm disaster.  Philippines: typhoons Haiyan and Washi | China: increased depression and PTSD symptoms.  Philippines: increased PTSD symptoms. Typhoon-related traumatic memories were associated with increased acute stress and depression symptoms. |
| 2 | 34 | No criteria | Pacific Islands | Pacific Islands | Climate change-related migration (e.g., loss of homeland due to sea level rise) | Decline in economic, mental, social, and community well-being |
| 3 | 31 | Subsistence Fishing communities | Micronesia | Micronesia | Non-specific climate change events (e.g., changes in the ecosystem due to ocean warming) | Decline in economic, mental, social, and community well-being |
| 4 | 50 | Indigenous populations | Global | Australia | Droughts, climate change-related landscape/homeland changes | Decline in economic, mental, social, and community well-being |
| 5 | 167 | Farmers | Global | Australia | Climate variability (e.g., droughts) | Decline in economic and mental well-being |
| 6 | 196 | No criteria | China | China | Floods, Typhoons/Cyclones | Increase in PTSD symptoms |
| 7 | - | Rural populations | Asia | China,  Philippines  Republic of Korea | China: Floods  Philippines: Typhoon Hayian.  Republic of Korea: Typhoon Russa | China, Philippines & Republic of Korea: increase in PTSD symptoms |
| 8 | 16 | Rural men aged more than 65 years old | Australia | Australia | Changes in a rural environment (e.g., because of prolonged droughts) | Increase in factors that induce a higher risk of suicide (e.g., increased debts, fewer job opportunities) |
| 9 | 17 | No criteria | Global | China  Vietnam | China: Flood, storm, snowstorm.  Vietnam: 2006 typhoon Xangsan | China: increased PTSD symptoms  Vietnam: increased PTSD, depression, and anxiety symptoms |
| 10 | 83 | No criteria | Global | Australia  China | Australia & China: Floods | Australia: increased tobacco, alcohol, substance, and medication use. There was no influence on depression symptoms of older adults or suicide rates.  China: increased PTSD symptoms |

Please note that this information was extracted when the authors explicitly reported findings of a given country/region of WPR in their text.

PTSD: Post Traumatic Stress Disorder

1: Sharpe, I., & Davison, C. M. (2022). A Scoping Review of Climate Change, Climate-Related Disasters, and Mental Disorders among Children in Low-and Middle-Income Countries. *International Journal of Environmental Research and Public Health*, *19*(5), 2896. <https://doi.org/10.3390/ijerph19052896>

2: Yates, O. E. T., Manuela, S., Neef, A., & Groot, S. (2022). Reshaping ties to land: a systematic review of the psychosocial and cultural impacts of Pacific climate-related mobility. *Climate and Development*, *14*(3), 250–267. <https://doi.org/10.1080/17565529.2021.1911775>

3: Hodgson, L., Fernando, G., & Lansbury, N. (2022). Exploring the Health Impacts of Climate Change in Subsistence Fishing Communities throughout Micronesia: A Narrative Review. *Weather, Climate, and Society*, *14*(3), 653–669. <https://doi.org/10.1175/WCAS-D-21-0169.1>

4: Middleton, J., Ashlee Cunsolo, Andria Jones-Bitton1, C. J. W. and, & Harper, S. L. (2020). Indigenous mental health in a changing climate: a systematic scoping review of the global literature. *Environmental Research Letters*, *15*(5). <https://doi.org/10.1088/1748-9326/ab68a9>

5: Daghagh Yazd, S., Ann Wheeler, S., & Zuo, A. (2019). Key Risk Factors Affecting Farmers' Mental Health: A Systematic Review. *International Journal of Environmental Research and Public Health*, *16*(23), 4849. <https://doi.org/10.3390/ijerph16234849>

6: Chan, E. Y. Y., Ho, J. Y., Hung, H. H. Y., Liu, S., & Lam, H. C. Y. (2019). Health impact of climate change in cities of middle-income countries: the case of China. *British Medical Bulletin*, *130*(1), 5–24. <https://doi.org/10.1093/bmb/ldz011>

7: Chan, E. Y. Y., Man, A. Y. T., & Lam, H. C. Y. (2019). Scientific evidence on natural disasters and health emergency and disaster risk management in Asian rural-based area. *British Medical Bulletin*, *129*(1), 91–105. <https://doi.org/10.1093/bmb/ldz002>

8: Crnek-Georgeson, K. T., Wilson, L. A., & Page, A. (2017). Factors influencing suicide in older rural males: a review of Australian studies. *Rural and Remote Health*, *17*(4), 4020. <https://doi.org/10.22605/RRH4020>

9: Rataj, E., Kunzweiler, K., & Garthus-Niegel, S. (2016). Extreme weather events in developing countries and related injuries and mental health disorders-a systematic review. *BMC Public Health*, *16*, 1020. <https://doi.org/10.1186/s12889-016-3692-7>

10: Fernandez, A., Black, J., Jones, M., Wilson, L., Salvador-Carulla, L., Astell-Burt, T., & Black, D. (2015). Flooding and mental health: A systematic mapping review. *PLoS ONE*, *10*(4), 1–20. <https://doi.org/10.1371/journal.pone.0119929>
